# Trends in Obesity Prevalence Among Patients Enrolled in Clinical Trials for Obesity-Related Cancers, 1986 to 2016

**DOI:** 10.1001/jamanetworkopen.2022.34445

**Published:** 2022-10-10

**Authors:** Riha Vaidya, Cathee Till, Heather Greenlee, Dawn L. Hershman, Joseph M. Unger

**Affiliations:** 1 SWOG Statistics and Data Management Center, Seattle, Washington; 2 Fred Hutchinson Cancer Center, Seattle, Washington; 3 Columbia University, New York, New York

## Abstract

**Importance:**

Obesity rates have risen in the United States since the 1980s. Several studies have shown links between obesity and the incidence of specific cancer types, but none have systematically examined obesity prevalence at the time of cancer diagnosis, or among clinical trial participants, all of which may affect clinical outcomes.

**Objective:**

To examine the prevalence of obesity and, separately, overweight and obesity over 30 years among patients with cancer enrolled in clinical treatment trials for obesity-related cancers; and to compare trends with corresponding trends in the US.

**Design, Setting, and Participants:**

This cohort study examined clinical treatment trials for obesity-related cancers conducted by the SWOG Cancer Research Network at community and academic sites. Participants included adult patients enrolled in phase 2 or phase 3 clinical treatment trials in obesity-related cancers between 1986 and 2016. Statistical analysis was performed from June 2020 to July 2022.

**Exposures:**

Year of enrollment to a clinical trial.

**Main Outcomes and Measures:**

Prevalence of obesity (body mass index [BMI] ≥ 30) and overweight or obesity (BMI > 25) at the time of clinical trial enrollment. Multivariable logistic regression analysis, adjusted for demographic and clinical factors, was used to analyze patient height and weight collected at clinical trial enrollment.

**Results:**

Among 23 926 patients (median [IQR] age, 58 [51-66] years; 17 594 [73.5%] female; 969 [4.0%] Hispanic, 2173 [9.1%] non-Hispanic Black, 19 890 [83.1%] non-Hispanic White) enrolled between 1986 and 2016, unadjusted obesity rates increased from 23.5% (in 1986 to 1990 [n = 657]) to 42.3% (in 2011 to 2016 [n = 836]). There was an increasing linear trend in obesity (odds ratio [OR], 1.23 for each 5-year increase; 95% CI, 1.21-1.26; *P* < .001), which persisted after covariate adjustment for demographic and clinical characteristics. Findings were consistent for the combined prevalence of overweight and obesity. The observed overall increasing trend in obesity prevalence from 1999-2000 to 2015-2016 was greater in trial patients (21.3% [SE = 0.7] to 49.1% [SE = 1.6]) than in US adults (30.5% [SE = 1.5] to 39.6% [SE = 1.6]) (*P *for trend = .03), but was similar to US cancer survivors (18.9% [SE = 1.9] to 42.2% [SE = 2.1]; *P *for trend = .31).

**Conclusions and Relevance:**

This cohort study found that patients with cancer and obesity are currently well-represented in cancer clinical trials, increasing confidence that trial findings are generalizable to patients with obesity in clinical practice. Availability of data on height and weight at diagnosis through cancer registries will enable a more rigorous analysis of obesity prevalence among patients with cancer.

## Key Points

**Question:**

What is the prevalence of obesity over time among patients with obesity-related cancers participating in clinical treatment trials?

**Findings:**

In this cohort study of 23 926 patients enrolled in phase 2 or phase 3 clinical treatment trials between 1986 and 2016, obesity prevalence at baseline during the years 1986 to 1990 was 23.5% and increased to 42.3% in 2011 to 2016, a statistically significant increasing linear trend that persisted after adjustment for demographic and clinical factors.

**Meaning:**

The increasing prevalence of obesity in clinical trial participants reflects similar patterns among US cancer survivors, suggesting that patients with obesity are well-represented in clinical trials for obesity-related cancers.

## Teaser

This cohort study investigates the prevalence of obesity and, separately, overweight and obesity over 30 years among patients enrolled in clinical treatment trials for obesity-related cancers compared with the overall US obesity trends.

## Introduction

Rising prevalence of obesity and overweight has been observed in the adult population in the United States since the 1980s.^1,2^ In recent years, obesity prevalence has exceeded 30% in most age and sex groups for adults,^3^ with prevalence among all adults reaching 38.9% in the period 2013 to 2016.^4^ A large body of evidence links obesity with the incidence of several types of cancer, including more common cancers such as breast and colorectal cancer.^5,6,7,8,9^ In 2014, obesity-related cancers accounted for 40% of cancer diagnoses.^10^ Along with being a risk factor for cancer incidence,^11^ obesity can also be associated with survival and recurrence,^12,13,14^ and of development of treatment toxic effects.^15,16^ While the mechanisms underlying these associations are not fully understood, several biological factors have been examined including insulin and insulin-like growth factor pathway, adipokines, sex steroids such as estrogen, and inflammation.^11,12,17^ Body size, in the form of weight or body surface area (BSA), also plays an important role in determining appropriate treatment dosage as some therapies require weight-based dosage,^18,19^ whereas a fixed dose is more appropriate for others.^20^

Despite the influence of obesity on cancer, prior research has only examined nationwide trends in obesity among individuals with a history of cancer.^21^ However, to our knowledge, no research has characterized the prevalence of obesity at cancer diagnosis. Moreover, the extent to which patients with obesity are represented in clinical trials has not been previously determined. The examination of obesity rates among cancer clinical trial participants is important for multiple reasons. First, clinical trials assess the safety and efficacy of new therapies before they are used in clinical practice settings and are used to determine dosage levels for therapies. Proportionate representation of patients with overweight and obesity in clinical trials is essential to help ensure that patient outcomes are examined in, and applicable to, these patients and to improve confidence about the generalizability of trial findings. Second, obesity is a risk factor for comorbid conditions which may pose a barrier to patient enrollment in clinical trials.^22^ Thus, examining prevalence of obesity among patients enrolled in cancer clinical trials may help highlight barriers to clinical trial participation. In this study, we examined the prevalence of obesity and overweight over a 30-year period among a large sample of patients enrolled in clinical treatment trials conducted in a diverse panel of obesity-related cancers.

## Methods

### Clinical Trial Data

We obtained data from clinical treatment trials conducted by the SWOG Cancer Research Network, a member of the National Cancer Institute’s (NCI’s) National Clinical Trials Network and the NCI’s Community Oncology Research Program. We systematically identified patients registered to phase 2 or phase 3 treatment trials for obesity-related cancers^23^ conducted between 1986 and 2016 that collected height and weight at enrollment. Obesity-related cancers are those for which there is sufficient evidence that obesity is related to an increased risk of developing the cancer; these include cancers of the breast (postmenopausal only), colon, endometrium, esophagus, gallbladder, gastric cardia, liver, ovaries, pancreas, rectum, kidney, and thyroid as well as meningioma and multiple myeloma.^23^ For breast cancer trials, menopausal status was determined using study registration data to identify postmenopausal patients. Each trial included in this secondary analysis was previously approved by an institutional review board; informed consent was previously obtained from all patients for each study. Institutional review board approval and informed consent of study participants for this cohort study was not required because secondary data that were not identifiable were used. We followed the Strengthening the Reporting of Observational Studies in Epidemiology (STROBE) reporting guideline for cohort studies.

### End Points

Baseline data on height and weight were obtained from trial case-report forms. Body mass index (BMI) was calculated as weight in kilograms divided by height in meters squared and classified as underweight (BMI < 18.5), normal weight (BMI 18.5-24.9), overweight (BMI 25.0-29.9) and obese (BMI ≥ 30). The primary end point was a binary variable indicating whether a patient had obesity at trial registration (yes vs no). For the secondary analysis, we used a binary variable indicating whether a patient was categorized as overweight or obese (BMI ≥ 25) at registration.

### Covariates

The main independent variable was year of registration to the clinical trial. To examine trends in obesity across demographic and clinical characteristics, we included age, sex, race, ethnicity, cancer type, the type of treatment the patient was assigned to in the clinical trial, and disease stage as independent variables. Age was used as a continuous variable. Race and ethnicity data were combined and characterized as Hispanic, non-Hispanic Black, non-Hispanic White, and other (which included Asian, Native Hawaiian or Pacific Islander, American Indian or Alaska Native, and multiracial). Race and ethnicity were self-reported and collected as part of routine clinical trial enrollment procedure. Cancer types were grouped as breast, gastrointestinal (cancers of the colon, esophagus, gallbladder, gastric cardia, liver, biliary ducts, pancreas, and rectum), gynecologic (endometrial and ovarian cancers), kidney, and multiple myeloma. Treatment types were classified as chemotherapy (with or without radiation), targeted therapy, biologic or immunotherapy, and combined systemic therapy. Hormone therapies for breast cancer (ie, tamoxifen in this data set) were classified as targeted therapy.^24^ To apply a consistent definition of disease stage across a broad set of cancers, study-level stage was categorized as advanced (2-year overall survival <75%) vs adjuvant.

### Statistical Analysis

To examine trends in obesity over 3 decades, time was categorized into consecutive 5-year periods (1986-1990, 1991-1995, 1996-2000, 2001-2005, 2006-2010, 2011-2016) based on year of trial registration. Calendar year 2016 was grouped with the 2011-to-2015 period. Logistic regression models were used to examine whether the odds of obesity differed over time, with time included as a continuous (ordered categorical) variable. The regression model was adjusted for demographic and clinical characteristics described previously. Interaction tests were used to examine whether time trends differed by categories of sex, race and ethnicity, disease stage, cancer type, and treatment type. Unadjusted and adjusted time trends within these categories were also examined using logistic regression.

Statistical tests were from Wald χ^2^ test statistics from multivariable logistic regression models. Analyses were conducted using SAS version 9.4 (SAS Institute) and R version 3.4.1 (R Project for Statistical Computing) from June 2020 to July 2022. We reported *P* < .05 as statistically significant.

To compare obesity prevalence in our data set alongside US adult obesity trends, we calculated obesity rates age standardized by the direct method to the US Census for the year 2000 for age groups 20 to 39 years, 40 to 59 years, and 60 years and over.^25^ Trial obesity rates were calculated for 2-year intervals from 1999-2000 to 2015-2016 for comparability with the published National Health and Nutrition Examination Survey (NHANES) obesity rates for all adults.^26^ We also calculated age-adjusted obesity rates among NHANES respondents who had ever been diagnosed with an obesity-related cancer (eMethods in the Supplement). The rates for the 3 groups were graphically displayed as smoothed lines using locally weighted smoothing.

To evaluate potential differences in obesity prevalence trends between groups, we derived coefficients representing the linear trajectory over time of the US adult and cancer survivor obesity prevalence rates, respectively, using linear regression. In separate linear regressions, we then assessed whether the trend for clinical trial patients differed by specifying the coefficients from the US adult and cancer survivor obesity prevalence trends, respectively, as the null hypotheses. Trends in the combined prevalence of obesity and overweight were examined as a secondary analysis.

## Results

Among the 23 926 patients who enrolled in phase 2 or phase 3 treatment trials (eTable 1 in the Supplement) for obesity-related cancers between 1986 and 2016 and were included in the study, 17 594 (73.5%) were female, 969 (4.0%) were Hispanic, 2173 (9.1%) were non-Hispanic Black, and 19 890 (83.1%) were non-Hispanic White; 12 547 patients (52.4%) were diagnosed with breast cancer and 7227 (30.2%) were enrolled in studies for advanced stage cancers; median (IQR) age was 58 (51-66) years (Table 1). Patient demographic and clinical characteristics varied by period of registration. The proportion of patients aged 60 years or older increased from 39.1% (n = 1091) between 1986 and 1990 to 51.3% (n = 1013) between 2011 and 2016, as did the proportion of Hispanic patients (1.0% [n = 27] between 1986 and 1990 to 8.1% [n = 160] between 2011 and 2016). More than a quarter of patients were enrolled in chemotherapy trials after 2005, with an increasing proportion enrolled in targeted therapy trials. The proportion of patients in trials for advanced stage disease increased from 26% (n = 726) in 1986-1990 to 54.9% (n = 1270) in 2001-2005 but decreased in more recent years.

**Table 1.** Participant Characteristics at SWOG Trial Registration

Characteristic	Participants, No. (%)						
	Overall	1986-1990	1991-1995	1996-2000	2001-2005	2006-2010	2011-2016
No.	23 926	2793	6512	4848	2315	5483	1975
Age, y							
18-39	1409 (5.9)	256 (9.2)	604 (9.3)	341 (7.0)	56 (2.4)	80 (1.5)	72 (3.6)
40-59	11 963 (50.0)	1446 (51.8)	3268 (50.2)	2427 (50.1)	1081 (46.7)	2851 (52.0)	890 (45.1)
≥60	10 554 (44.1)	1091 (39.1)	2640 (40.5)	2080 (42.9)	1178 (50.9)	2552 (46.5)	1013 (51.3)
Sex							
Female	17 594 (73.5)	2337 (83.7)	4963 (76.2)	2635 (54.4)	1545 (66.7)	5046 (92.0)	1068 (54.1)
Male	6332 (26.5)	456 (16.3)	1549 (23.8)	2213 (45.6)	770 (33.3)	437 (8.0)	907 (45.9)
Race and ethnicity							
Hispanic	969 (4.0)	27 (1.0)	282 (4.3)	174 (3.6)	100 (4.3)	226 (4.1)	160 (8.1)
Non-Hispanic							
Black	2173 (9.1)	258 (9.2)	692 (10.6)	420 (8.7)	184 (7.9)	409 (7.5)	210 (10.6)
White	19 890 (83.1)	2416 (86.5)	5347 (82.1)	4102 (84.6)	1924 (83.1)	4597 (83.8)	1504 (76.2)
Other^a^	894 (3.7)	92 (3.3)	191 (2.9)	152 (3.1)	107 (4.6)	251 (4.6)	101 (5.1)
Treatment type							
Chemotherapy	14 658 (61.3)	1984 (71.0)	4281 (65.7)	4786 (98.7)	1598 (69.0)	1444 (26.3)	565 (28.6)
Biologic/immunotherapy	646 (2.7)	19 (0.7)	206 (3.2)	37 (0.8)	75 (3.2)	197 (3.6)	112 (5.7)
Targeted therapy	5277 (22.1)	93 (3.3)	279 (4.3)	25 (0.5)	243 (10.5)	3548 (64.7)	1089 (55.1)
Combination systemic therapy	3345 (14.0)	697 (25.0)	1746 (26.8)	NA	399 (17.2)	294 (5.4)	209 (10.6)
Cancer type							
Breast	12 547 (52.4)	2067 (74.0)	3775 (58.0)	605 (12.5)	784 (33.9)	4792 (87.4)	524 (26.5)
Gastrointestinal	6506 (27.2)	351 (12.6)	1493 (22.9)	2944 (60.7)	1064 (46.0)	352 (6.4)	302 (15.3)
Gynecological	1054 (4.4)	3 (0.1)	243 (3.7)	559 (11.5)	245 (10.6)	4 (0.1)	NA
Multiple myeloma	2366 (9.9)	273 (9.8)	656 (10.1)	703 (14.5)	40 (1.7)	335 (6.1)	359 (18.2)
Kidney	1453 (6.1)	99 (3.5)	345 (5.3)	37 (0.8)	182 (7.9)	NA	790 (40.0)
Study stage							
Advanced	7227 (30.2)	726 (26.0)	2096 (32.2)	1770 (36.5)	1270 (54.9)	807 (14.7)	558 (28.3)
Adjuvant	16 699 (69.8)	2067 (74.0)	4416 (67.8)	3078 (63.5)	1045 (45.1)	4676 (85.3)	1417 (71.7)

Abbreviaton: NA, not available (no observations).

^a^ The other category included patients who reported non-Hispanic ethnicity and 1 of the following races: Asian, Native Hawaiian or Pacific Islander, American Indian or Alaska Native, multiracial.

### Unadjusted Obesity and Overweight Prevalence

**Figure 1.**
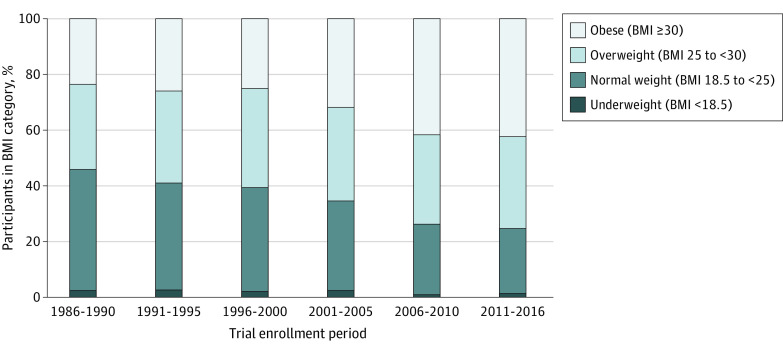
Proportion of Participants in SWOG Clinical Trials Over Time by BMI Category The bars show percentage of clinical trial participants in each BMI category for each 5-year period of trial enrollment. BMI indicates body mass index (calculated as weight in kilograms divided by height in meters squared).

Over time, we observed increasing prevalence of obesity among patients enrolled in trials which corresponded with a decrease in the proportion of patients in the normal weight category (Figure 1). The proportion of patients with obesity increased from 23.5% (n = 657) in 1986 to 1990 to 42.3% (n = 836) in 2011 to 2016. Although prevalence of overweight increased from 30.7% (n = 857) in 1986to 1990 to 35.6% (n = 1725) in 1996 to 2000, it decreased slightly in subsequent years and was at 33% (n = 652) in 2011 to 2016.

**Figure 2.**
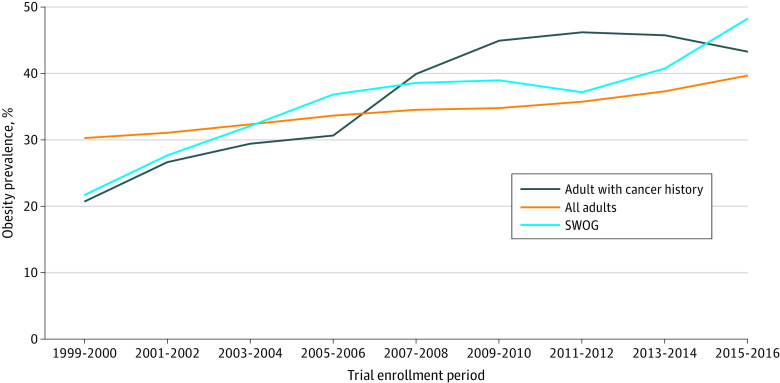
Age-Standardized Obesity Prevalence Over Time for US Adults and for Adults Enrolled in SWOG Clinical Trials Rates are expressed as percentages and standardized to the age distribution for the US population for the year 2000. US adult obesity rates are published rates based on the National Health and Nutrition Examine Surveys (NHANES); obesity rates for US adults with a history of obesity-related cancer were calculated using NHANES; SWOG rates have been calculated using the SWOG trial database used in this analysis.

From 1986-1990 to 2011-2016, obesity prevalence increased from 24.7% (95% CI, 23.0%-26.5%) to 44.8% (95% CI, 41.8%-47.7%) (*P* < .001) for female patients and from 17.3% (95% CI, 13.8%-20.8%) to 39.5% (95% CI, 36.3%-42.7%) (*P* < .001) for male patients (Table 2). Obesity rates were highest among non-Hispanic Black patients. Obesity prevalence increased across subgroups by disease, treatment, and stage over time (Table 2 and Figure 2). The combined prevalence of overweight and obesity increased from 54.2% from 1986-1990 to 75.3% from 2011-2016 (eTable 2 in the Supplement).

**Table 2.** Obesity Prevalence Rates

Characteristic	OR (95% CI)					
	1986-1990	1991-1995	1996-2000	2001-2005	2006-2010	2011-2016
No.	2793	6512	4848	2315	5483	1975
Overall	23.5 (21.9 to 25.1)	25.9 (24.8 to 27.0)	25.0 (23.8 to 26.2)	31.9 (30.0 to 33.8)	41.5 (40.2 to 42.9)	42.3 (40.1 to 44.5)
Sex						
Female	24.7 (23.0 to 26.5)	28.6 (27.3 to 29.8)	26.5 (24.8 to 28.1)	37.0 (34.6 to 39.4)	42.1 (40.8 to 43.5)	44.8 (41.8 to 47.7)
Male	17.3 (13.8 to 20.8)	17.3 (15.4 to 19.2)	23.3 (21.6 to 25.1)	21.6 (18.6 to 24.5)	34.8 (30.3 to 39.3)	39.5 (36.3 to 42.7)
Race and ethnicity						
Hispanic	25.9 (8.3 to 43.6)	26.2 (21.1 to 31.4)	24.7 (18.2 to 31.2)	37.0 (27.4 to 46.6)	43.4 (36.9 to 49.9)	40.0 (32.3 to 47.7)
Non-Hispanic						
Black	40.7 (34.7 to 46.7)	35.8 (32.3 to 39.4)	30.0 (25.6 to 34.4)	38.0 (31.0 to 45.1)	59.9 (55.1 to 64.7)	52.4 (45.6 to 59.2)
White	21.9 (20.3 to 23.6)	25.0 (23.8 to 26.2)	25.0 (23.6 to 26.3)	31.7 (29.6 to 33.7)	40.4 (39.0 to 41.8)	42.4 (39.9 to 44.9)
Other	16.3 (8.6 to 24.0)	14.7 (9.6 to 19.7)	13.2 (7.7 to 18.6)	20.6 (12.8 to 28.3)	31.1 (25.3 to 36.8)	24.8 (16.2 to 33.3)
Treatment						
Chemotherapy	21.5 (19.7 to 23.3)	21.7 (20.5 to 22.9)	25.0 (23.7 to 26.2)	35.7 (33.4 to 38.1)	45.7 (43.1 to 48.3)	43.2 (39.1 to 47.3)
Biologic/immunotherapy	31.6 (8.6 to 54.6)	20.9 (15.3 to 26.5)	27.0 (12.0 to 42.0)	30.7 (20.0 to 41.3)	38.1 (31.2 to 44.9)	50.9 (41.5 to 60.3)
Targeted therapy	36.6 (26.6 to 46.5)	40.5 (34.7 to 46.3)	32.0 (12.3 to 51.7)	27.6 (21.9 to 33.2)	40.5 (38.9 to 42.1)	41.1 (38.2 to 44.1)
Combined systemic therapy	27.4 (24.1 to 30.7)	34.5 (32.2 to 36.7)		19.3 (15.4 to 23.2)	36.1 (30.5 to 41.6)	41.6 (34.9 to 48.4)
Cancer type						
Breast	25.5 (23.7 to 27.4)	30.5 (29.0 to 31.9)	29.1 (25.5 to 32.7)	46.9 (43.4 to 50.4)	42.5 (41.1 to 43.9)	47.5 (43.2 to 51.8)
Gastrointestinal	15.4 (11.6 to 19.2)	16.9 (15.0 to 18.9)	23.6 (22.1 to 25.2)	20.1 (17.7 to 22.5)	29.3 (24.5 to 34.0)	23.8 (19.0 to 28.7)
Gynecological		23.0 (17.7 to 28.4)	22.7 (19.2 to 26.2)	27.3 (21.7 to 33.0)	50.0 (−41.9 to 141.9)	
Multiple myeloma	19.0 (14.4 to 23.7)	22.6 (19.4 to 25.8)	29.0 (25.7 to 32.4)	45.0 (28.9 to 61.1)	40.3 (35.0 to 45.6)	45.1 (40.0 to 50.3)
Kidney	23.2 (14.8 to 31.7)	23.2 (18.7 to 27.7)	27.0 (12.0 to 42.0)	39.0 (31.9 to 46.2)		44.7 (41.2 to 48.2)
Study stage						
Advanced	17.8 (15.0 to 20.6)	18.9 (17.2 to 20.6)	25.0 (23.0 to 27.0)	22.8 (20.5 to 25.1)	35.7 (32.4 to 39.0)	30.5 (26.6 to 34.3)
Adjuvant	25.5 (23.7 to 27.4)	29.2 (27.9 to 30.6)	25.0 (23.5 to 26.5)	42.9 (39.9 to 45.9)	42.6 (41.1 to 44.0)	47.0 (44.4 to 49.6)

Abbreviation: OR, odds ratio.

Between 1999-2000 and 2015-2016, age-adjusted obesity rates increased from 30.5% (SE = 1.5) in 1999-2000 to 39.6% (SE = 1.6) in 2015-2016 among all adults, and from 18.9% (SE = 1.9) to 42.2% (SE = 2.1) among cancer survivors. In comparison, among trial participants, prevalence increased from 21.3% (SE = 0.7) to 49.1% (SE = 1.6) over the same period. Obesity rates among SWOG patients were lower than rates for all US adults at the beginning of the period, but then exceeded rates for all adults from 2003-2004 onward. The trend toward increasing prevalence of obesity in trial participants compared to all adults was statistically significant (*P* for trend = .03). In contrast, obesity prevalence trends in trial participants more closely reflected those in US cancer survivors, with no difference in trends overall (*P* for trend = .31).

### Model-Adjusted Obesity Trends

**Figure 3.**
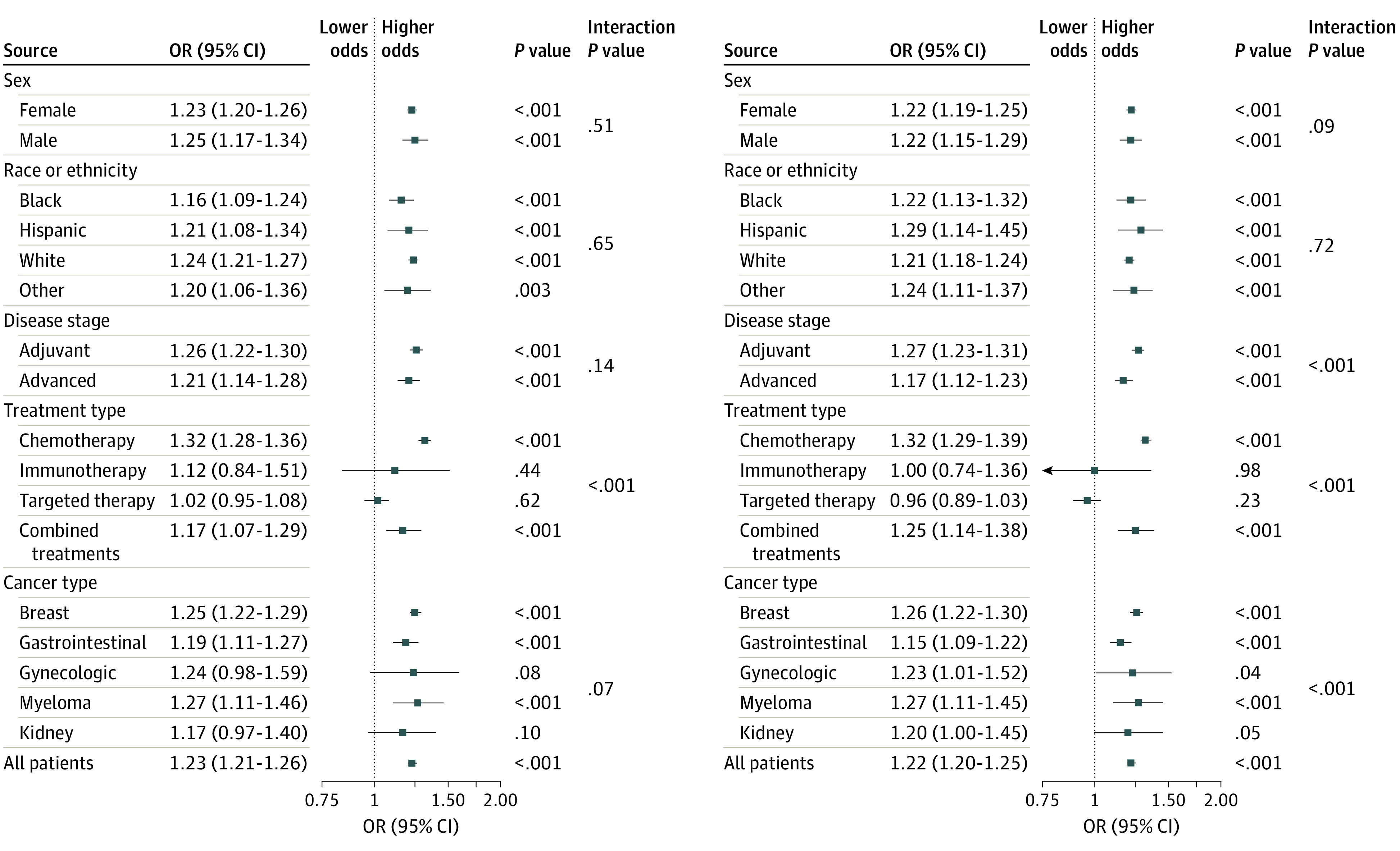
Forest Plot of the Association of Obesity Prevalence and Combined Overweight and Obesity Prevalence With Trial Enrollment Period The boxes indicate the odds ratio and the horizontal lines indicate the 95% CI. Odds ratio, 95% CI, and *P* value calculated using logistic regression adjusted for age, sex, race and ethnicity, cancer type, stage, and treatment. Interaction *P* value tests interaction between registration year and stratification factor. Boxes to the right of the vertical line (the line of equal odds) indicate increased risk of the outcome over time.

For all patients, there was a statistically significant increasing linear trend in obesity (OR = 1.23 per 5-year increase, 95% CI: 1.21-1.26, *P* < .001) (Figure 3). We observed statistically significant interactions between time and cancer treatment type (*P *for interaction < .001) indicating that obesity trends differed by treatment.

In subgroup analyses, odds of obesity increased over time for both female participants (OR, 1.23 per 5-year increase; 95% CI, 1.20-1.26, *P* < .001) and male participants (OR, 1.25 per 5-year increase; 95% CI, 1.17-1.34; *P* < .001). Increasing trends in obesity were also observed for patients who received chemotherapy or combined systemic therapy, but not for targeted therapy or immunotherapy (Figure 3). Increasing odds of obesity were observed for breast cancer, gastrointestinal cancers, and multiple myeloma, for patients of all racial and ethnic groups, and for patients in both advanced and adjuvant studies (Figure 3).

### Obesity and Overweight Trends

Our secondary analysis showed a statistically significant increasing linear trend in the combined prevalence of overweight and obesity (OR, 1.22 per 5-year increase; 95% CI, 1.20-1.25; *P* < .001). The odds of overweight/obesity over time differed by cancer type, study stage, and treatment (all *P* for interaction < .001), but not by sex (*P* for interaction = .09) (Figure 3) or race and ethnicity (*P* for interaction = .72). A statistically significant increasing linear trend in combined overweight and obesity prevalence was observed for all subgroups, except patients in the targeted therapy and immunotherapy groups and patients with kidney cancer (Figure 3).

## Discussion

In this study, we examined trends in obesity at diagnosis among patients with obesity-related cancers using pooled data from clinical trials conducted by an NCI-supported national cancer research network. Over a 30-year period, obesity prevalence increased substantially. These trends persisted after covariate adjustment for key demographic and clinical characteristics and were observed in nearly all included cancer types and all racial and ethnic groups. Overall increases in obesity prevalence rates among clinical trial participants also exceeded corresponding increases in the US adult general population over a similar period but were similar to trends among cancer survivors in the general population.

Increasing obesity among adults in the United States from the 1980s to recent years has been well described.^1,2^ Obesity prevalence among cancer survivors has been examined in nationally representative data^21^ and has been found to exceed obesity prevalence in the general population. However, importantly, there are no known national estimates of obesity prevalence among adult patients with cancer at the time of diagnosis, either in trials or outside of trials. Clinical trials, which routinely collect several types of patient data, also do not routinely report enrollment of patients with obesity.^27^ In a systematic review and analysis of cancer clinical trials, Pestine et al^27^ were able to analyze published or unpublished obesity data for less than a third of the 76 randomized clinical trials for obesity-related cancers included in their study. By pooling a diverse set of obesity -related cancers over a 30-year period, we were able to determine that patients with obesity are currently well-represented in NCI-supported clinical trials.

In our analysis of obesity trends by cancer type and treatment, we did not find significant trends in prevalence for gynecologic and kidney cancers, and for patients receiving immunotherapy. For gynecologic and kidney cancers, relatively small sample sizes may have limited our ability to detect a trend. In unadjusted models, we observed a significant increasing trend in obesity for patients treated with immunotherapy, which was no longer statistically significant after adjustment for covariates. Thus, the absence of a statistically significant trend over time in obesity prevalence among immunotherapy-treated patients may have been driven by the limited cancer types and amount of time for which patients were treated with immunotherapy in our data set.

Metabolic changes caused by obesity have been studied and implicated in cancer incidence^17^ and may also play a role in the poor prognosis^13^ and worse survival outcomes^28^ associated with obesity for commonly occurring cancers. These biological mechanisms could potentiate the development of obesity-related cancers, such that increasing rates of obesity in the population at large may lead to an accelerating proportion of patients with cancer and obesity. In this context, recent findings by Koroukian et al^29^ showed a shift toward younger age groups in obesity-related cancers, consistent with the outsized increase in obesity prevalence in younger individuals. Thus, we would expect obesity trends among patients with obesity-related cancers to differ substantially from those in the general population. Consistent with this, we found that obesity prevalence trends among clinical trial participants were increasing at a faster rate than those in the US adult population but not faster than those among cancer survivors. This observation supports the literature describing the role of obesity in the development of cancer, and also reinforces prior observations that the burden of obesity among cancer survivors is increasing. However, these findings are limited by the absence of prediagnosis BMI data for trial participants and by the absence of BMI data at diagnosis for the cancer survivor population in the NHANES data set.

The disease sites and stage represented in our data set are a function of the clinical trials that were open to enrollment between 1986 and 2016 and may have limited generalizability. For instance, fewer trials for advanced stage disease in our sample could result in inadequate accounting for cachexia experienced by patients with advanced disease^13^; if so, our results could overestimate obesity prevalence. Fortunately, the validity of our findings was improved by the observation of increasing obesity prevalence for patients with both advanced and adjuvant stage disease; furthermore, there was no evidence of differences in trends by disease stage.

Findings from randomized clinical treatment trials play an important role in the development of clinical practice guidelines, including determination of optimal dose levels and drug combinations.^30^ Our findings indicate that patients with obesity are well-represented in trials, improving confidence that newly proven cancer therapies for obesity-related cancers will be generalizable to patients with cancer and obesity. This is consistent with recent ASCO guidelines recommending full weight-based dosing of chemotherapy and full approved dosing of checkpoint inhibitors and targeted therapies, instead of capping dosage by ideal weight as has been observed in clinical practice.^31^ Although evidence on the association between obesity and treatment toxic effects is mixed, increased cardiovascular toxic effects have been observed among patients with breast cancer and obesity treated with anthracyclines and/or trastuzumab.^31^ Higher BMI has also been found to be associated with increased immune-related toxic effects among patients treated with immune checkpoint inhibitors.^32^ Increasing obesity among trial participants provides an opportunity to further examine treatment toxic effects among patients with cancer and obesity. Finally, the increased burden of comorbidities associated with obesity^33^ may increase the risk of noncancer morbidity and morbidity among trial participants as obesity prevalence increases.

### Strengths and Limitations

This study has several strengths. To our knowledge, this is the first study to examine obesity prevalence among patients enrolled in clinical treatment trials for cancer. Our analysis used a large clinical trial database combining patient-level data from multiple clinical trials conducted across the United States over a 30-year period. We used height and weight data collected at trial registration which allowed us to examine obesity prevalence prior to administration of cancer therapy.

This study also had some limitations. Our database did not include trials for meningioma and thyroid cancer. We were unable to include trials without height and weight data. Our analysis did not include data from studies that are actively accruing patients or those that have not published any data by the time of this analysis, thus excluding more recent years (2017-2020) from the analysis. Furthermore, although it is commonly used, BMI is a limited measure of health and may not accurately estimate body fat levels which are implicated in cancer incidence.^6,17^ Finally, we were unable to examine the extent to which obesity prevalence in trial patients was representative of patients with cancer receiving active treatment outside of clinical trials, given the absence of normative data on obesity prevalence in the cancer population.

## Conclusions

Our study found that patients with obesity are currently well represented in clinical trials for obesity-related cancers, improving confidence that clinical trial findings are applicable to this set of patients. The consistent availability and reporting of height and weight data at diagnosis or treatment initiation for a broader set of patients with cancer through registries or other data sources would enable a more rigorous understanding of representation of obese patients in cancer clinical trials. Additionally, obesity prevalence rates among patients enrolled in SWOG clinical trials has been increasing over 30 years, reflecting broader trends in the US population and indicative of the increasing burden of obesity among patients with obesity-related cancers.

## Notes

Supplement. **eMethods.** Calculation of Obesity Rates Among NHANES Participants With a Cancer Diagnosis **eTable 1.** List of Studies Included in the Analysis **eTable 2.** Combined Overweight and Obesity Prevalence Rates (Percent)
