# Supplementary material for: Trends in Obesity Prevalence Among Patients Enrolled in Clinical Trials for Obesity-Related Cancers, 1986 to 2016
Source: JAMA Netw Open. 2022 Oct 10;5(10):e2234445. doi: 10.1001/jamanetworkopen.2022.34445 (PMC9552895; doi:10.1001/jamanetworkopen.2022.34445)
Supplement: Supplement. — eMethods. Calculation of Obesity Rates Among NHANES Participants With a Cancer Diagnosis eTable 1. List of Studies Included in the Analysis eTable 2. Combined Overweight and Obesity Prevalence Rates (Percent) [file jamanetwopen-e2234445-s001.pdf]

Supplemental Online Content

Vaidya R, Till C, Greenlee H, Hershman DL, Unger JM. Trends in obesity prevalence among patients enrolled in clinical trials for obesity-related cancers, 1986 to 2016. *JAMA Netw Open.* 2022;5(10):e2234445. doi:10.1001/jamanetworkopen.2022.34445

eMethods. Calculation of Obesity Rates Among NHANES Participants With a Cancer Diagnosis

eTable 1. List of Studies Included in the Analysis

eTable 2. Combined Overweight and Obesity Prevalence Rates (Percent)

This supplemental material has been provided by the authors to give readers additional information about their work.

© 2022 Vaidya R et al. *JAMA Network Open.*

eMethods: Calculation of obesity rates among NHANES participants with a cancer diagnosis

The National Health and Nutrition Examination Surveys (NHANES) data were used to calculate obesity prevalence among US adults with a history of obesity-related cancer. For comparability with the SWOG dataset as well as with published US adult obesity rates based on NHANES, we used survey cycles 1999-2000 to 2015-16. For each survey cycle, participants were included in the analysis if they were aged 20 years or older, were not pregnant at the time of the interview, and had height and weight data recorded in NHANES.

The NHANES medical conditions questionnaire was used to identify cancer history. Participants were asked if they had ever received a diagnosis of cancer and, if they had received a diagnosis, the type of cancer they were diagnosed with. Each participant could enter up to three types of cancer that they had been diagnosed with. For our analysis, we characterized participants as having a history of obesity-related cancer if any of their three diagnoses were for an obesity-related cancer.

Proportions of individuals with obesity among those with a history of obesity-related cancer were calculated. Survey weights provided in the NHANES datasets were applied to account for the complex stratified sampling of the survey and to ensure that estimated proportions were nationally representative.

© 2022 Vaidya R et al. *JAMA Network Open.*

eTable 1: List of Studies Included in the Analysis

Cancer Type	Study ID
Breast	S8814, S8897, S9623, S9625, S0012, S0221, S0307, S0338, S0430, S0500, S0800
Gastrointestinal	S8614, S8905, S8910, S8916, S8917, S8933, S8936, S9008, S9051, S9060, S9100, S9107, S9135, S9150, S9241, S9304, S9339, S9413, S9415, S9420, S9923, S9924, S0030, S0101, S0107, S0127, S0202, S0205, S0336, S0356, S0413, S0414, S0415, S0514, S0600, S0713, S0941, S1005, S1115, S1310
Gynecologic	S8904, S8914, S9106, S9249, S9324, S9326, S9618, S9619, S9701, S9720, S9912, S0009
Multiple Myeloma	S8624, S8900, S9028, S9210, S9237, S9321, S0232, S0434, S0777, S1304
Renal	S8824, S8926, S8929, S8945, S8949, S9012, S9104, S9122, S9230, S9338, S0109, S0312, S0317, S0412, S0931, S1107

© 2022 Vaidya R et al. *JAMA Network Open.*

eTable 2: Combined Overweight and Obesity Prevalence Rates (Percent)

	1986-1990	1991-1995	1996-2000	2001-2005	2006-2010	2011-2016
	N=2793	N=6512	N=4848	N=2315	N=5483	N=1975
Overall	54.2 (52.4-56.1)	59.0 (57.8-60.2)	60.6 (59.2-62.0)	65.4 (63.4-67.3)	73.7 (72.5-74.8)	75.3 (73.4-77.2)
By Sex						
Female	54.1 (52.1-56.1)	60.0 (58.6-61.4)	57.3 (55.4-59.2)	68.0 (65.6-70.3)	73.8 (72.6-75.0)	72.8 (70.2-75.5)
Male	54.8 (50.2-59.4)	55.8 (53.3-58.3)	64.5 (62.5-66.5)	60.1 (56.7-63.6)	72.5 (68.3-76.7)	78.3 (75.6-81.0)
By Race/Ethnicity						
Hispanic	55.6 (35.5-75.6)	61.7 (56.0-67.4)	64.4 (57.2-71.6)	65.0 (55.5-74.5)	83.2 (78.3-88.1)	76.9 (70.3-83.5)
Non-Hispanic Black	68.2 (62.5-73.9)	64.6 (61.0-68.2)	66.0 (61.4-70.5)	71.2 (64.6-77.8)	83.6 (80.0-87.2)	84.3 (79.3-89.2)
Non-Hispanic White	53.3 (51.3-55.3)	58.8 (57.5-60.1)	60.6 (59.1-62.1)	65.5 (63.4-67.6)	72.9 (71.6-74.2)	75.0 (72.8-77.2)
Other	39.1 (29.0-49.3)	40.8 (33.8-47.9)	41.4 (33.5-49.4)	53.3 (43.7-62.9)	63.3 (57.3-69.3)	59.4 (49.7-69.1)
By Treatment						
Chemotherapy	52.5 (50.3-54.7)	54.2 (52.7-55.7)	60.6 (59.3-62.0)	68.8 (66.6-71.1)	77.6 (75.5-79.8)	73.6 (70.0-77.3)
Biologic/Immunotherapy	42.1 (17.7-66.6)	55.8 (49.0-62.7)	51.4 (34.5-68.2)	58.7 (47.3-70.1)	70.6 (64.1-77.0)	74.1 (65.9-82.3)
Targeted Therapy	72.0 (62.8-81.3)	76.7 (71.7-81.7)	68.0 (48.3-87.7)	58.0 (51.8-64.3)	72.4 (70.9-73.9)	76.4 (73.9-78.9)
Combined Systemic Therapy	57.1 (53.4-60.8)	68.2 (66.0-70.4)		57.1 (52.3-62.0)	72.1 (67.0-77.3)	75.1 (69.2-81.0)
By Cancer Type						
Breast	54.6 (52.5-56.8)	62.6 (61.1-64.2)	59.3 (55.4-63.3)	78.1 (75.2-81.0)	74.2 (73.0-75.5)	77.7 (74.1-81.2)
Gastrointestinal	49.3 (44.0-54.5)	49.8 (47.3-52.4)	60.4 (58.6-62.1)	55.2 (52.2-58.2)	65.1 (60.1-70.1)	54.3 (48.7-60.0)
Gynecologic		53.5 (47.2-59.8)	51.3 (47.2-55.5)	60.8 (54.7-67.0)	100.0	
Multiple Myeloma	57.9 (52.0-63.8)	60.8 (57.1-64.6)	70.6 (67.2-73.9)	72.5 (58.0-87.0)	74.6 (69.9-79.3)	74.9 (70.4-79.4)
Renal	54.5 (44.6-64.5)	59.4 (54.2-64.6)	51.4 (34.5-68.2)	74.7 (68.4-81.1)		82.0 (79.3-84.7)
By Study Stage						
Advanced	53.0 (49.4-56.7)	52.8 (50.7-55.0)	60.7 (58.5-63.0)	58.3 (55.6-61.0)	68.8 (65.6-72.0)	62.0 (58.0-66.0)
Adjuvant	54.6 (52.5-56.8)	61.9 (60.5-63.4)	60.5 (58.8-62.3)	74.0 (71.3-76.6)	74.5 (73.3-75.8)	80.6 (78.5-82.7)

© 2022 Vaidya R et al. *JAMA Network Open.*
